# Developing Non-Laboratory Cardiovascular Risk Assessment Charts and Validating Laboratory and Non-Laboratory-Based Models

**DOI:** 10.5334/gh.890

**Published:** 2021-09-02

**Authors:** Razieh Hassannejad, Marjan Mansourian, Hamidreza Marateb, Mohammad Reza Mohebian, Thomas Andrew Gaziano, Rodney T. Jackson, Emanuele Di Angelantonio, Nizal Sarrafzadegan

**Affiliations:** 1 Isfahan Cardiovascular Research Center, Cardiovascular Research Institute, Isfahan University of Medical Sciences, Isfahan, IR; 2 Department of Epidemiology and Biostatistics, School of Public Health, Isfahan University of Medical Sciences, Isfahan, IR; 3 Department of Automatic Control, Biomedical Engineering Research Center, Universitat Politècnica de Catalunya, BarcelonaTech (UPC), Barcelona, ES; 4 Biomedical Engineering Department, Engineering faculty, University of Isfahan, Isfahan, IR; 5 Department of Electrical and Computer Engineering, University of Saskatchewan, Saskatoon, SK, S7N 5A9, CA; 6 Cardiovascular Medicine Division, Brigham and Women’s Hospital, Boston, US; 7 Epidemiology and Biostatistics Department, Population Health, Faculty of Medical and Health Sciences, University of Auckland, Auckland, NZ; 8 Cardiovascular Epidemiology Unit, Department of Public Health and Primary Care, University of Cambridge, Cambridge, UK; 9 School of Population and Public Health, Faculty of Medicine, University of British Columbia, Vancouver, British Columbia, CA

**Keywords:** Cardiovascular Disease, risk assessment, Laboratory-based model, Non-laboratory-based model, Isfahan Cohort Study

## Abstract

**Background:**

Developing simplified risk assessment model based on non-laboratory risk factors that could determine cardiovascular risk as accurately as laboratory-based one can be valuable, particularly in developing countries where there are limited resources.

**Objective:**

To develop a simplified non-laboratory cardiovascular disease risk assessment chart based on previously reported laboratory-based chart and evaluate internal and external validation, and recalibration of both risk models to assess the performance of risk scoring tools in other population.

**Methods:**

A 10-year non-laboratory-based risk prediction chart was developed for fatal and non-fatal CVD using Cox Proportional Hazard regression. Data from the Isfahan Cohort Study (ICS), a population-based study among 6504 adults aged ≥ 35 years, followed-up for at least ten years was used for the non-laboratory-based model derivation. Participants were followed up until the occurrence of CVD events. Tehran Lipid and Glucose Study (TLGS) data was used to evaluate the external validity of both non-laboratory and laboratory risk assessment models in other populations rather than one used in the model derivation.

**Results:**

The discrimination and calibration analysis of the non-laboratory model showed the following values of Harrell’s C: 0.73 (95% CI 0.71–0.74), and Nam-D’Agostino χ^2^:11.01 (p = 0.27), respectively. The non-laboratory model was in agreement and classified high risk and low risk patients as accurately as the laboratory one. Both non-laboratory and laboratory risk prediction models showed good discrimination in the external validation, with Harrell’s C of 0.77 (95% CI 0.75–0.78) and 0.78 (95% CI 0.76–0.79), respectively.

**Conclusions:**

Our simplified risk assessment model based on non-laboratory risk factors could determine cardiovascular risk as accurately as laboratory-based one. This approach can provide simple risk assessment tool where laboratory testing is unavailable, inconvenient, and costly.

## Introduction

Cardiovascular disease (CVD) is the most common preventable non-communicable diseases (NCD) worldwide, with an estimated 17.8 million deaths in 2017. It is predicted that CVD would be the cause of more than 23 million (about 30.5%) deaths by 2030 worldwide [1,2,3].

A reduction of CVD mortality rates has been reported in high income regions. However, 50% of CVD mortality and 80% of the CVD global burden occur in low and middle-income countries (LMICs), including the Eastern Mediterranean Region (EMR) [4].

In the last two decades, the most common causes of death have been transited from infectious to NCDs specially CVD in Iran [5]. Global Burden of Disease(GBD) previous data in 2010 and 2015 reported that CVD was the first leading cause of mortality and DALYs that led to 46% of all deaths and 20–23% of the burden of diseases in Iran [6].

Many global prevention and control guidelines recommend applying CVD risk assessment charts to identify people at high risk of developing CVD within a specified period of time, usually 10 years. Risk-based management helps to target specific intensive preventive and treatment interventions [7,8,9,10]. Several risk assessment models have been reported previously [11,12,13,14,15,16,17,18]. Although the widely used risk algorithms could be beneficial in developed countries, however, they are based on laboratory measures that are not affordable and available in some LMICs [19, 20].

Recently, significant efforts have been directed to develop non-laboratory based CV risk algorithms that can predict the disease as accurately as laboratory-based ones but are more feasible to use in clinical practice [10,20].

Laboratory and non-laboratory based CVD risk charts were developed by WHO for different regions worldwide and validated in other cohorts [10]. However, one potential limitation is the use of HRs derived in a mainly western population in a LMIC. Additionally, the new WHO charts are recalibrated for region and might be improved by focusing on national data. Providing affordable approaches for the prediction of CVD risk based on national data is especially crucial in LMICs, where many primary care facilities are not available, and most individuals remain unaware of their underlying cardiovascular risk [20,21].

In an attempt to simplify the Persian Atherosclerotic cardiovascular disease Risk Stratification (PARS) model that we reported previously and is a laboratory-based one [22], we aim to develop a simplified Persian Atherosclerotic cardiovascular disease Risk Stratification (SPARS) model based on non-laboratory risk factors and to assess if it can predict CVD risk as accurately as the PARS laboratory-based one. Furthermore, we aim to validate PARS and SPARS models, both developed based on Isfahan Cohort study(ICS) and to validate them on the Tehran Lipid and Glucose Study (TLGS) population [23].

## Methods

### Study population

The ICS is a population-based longitudinal study of 6504 Iranians adult, who were recruited in the year 2001 using multi-stage random cluster sampling and followed-up for at least ten years [24].

Written, informed consents were obtained from all subjects and Ethical approval was obtained from the Isfahan Cardiovascular Research Center Ethics Committee, a WHO collaborating center in the Eastern Mediterranean Region (EMR), and Isfahan University of Medical Sciences and conformed to the Declaration of Helsinki.

Having Iranian nationality, aged ≥ 35 years, mentally competent, and not pregnant, were considered as inclusion criteria. Participants with known coronary heart disease, heart failure, stroke and ischemic heart attack (n = 181) and subjects without a single follow-up (n = 891) were excluded from the study. Among the original recruited sample, only 5432 were free of CVD at baseline and had at least one follow-up.

Phone call follow-up was done every two years to look for any report of CVD events including sudden death, unstable angina, fatal and non-fatal myocardial infarction, and fatal and non-fatal stroke. All medical records of participants who reported CVD events were collected and verbal autopsies were done for dead participants. A panel of cardiologists and neurologists made final decisions by reviewing on the patient’s medical records and validated and confirmed the events diagnosis [25].

The loss to follow-up rate was 891 (14.1%) in the first phone call follow-ups and decrease to 104 (1.6%) in the fifth stage. The baseline characteristics and prevalence of CVD risk factors were not significantly different between subjects lost to follow-up and those studied [25,26]. Participants without any event and loss to follow-up events were considered as censored. The detailed description of the design, methodology, follow-up, success rate of follow-up, risk factor measurements, and endpoints of ICS were previously reported [22,25,27].

### External Cohort

The Tehran Lipid and Glucose Study (TLGS) was used for external validation because of the similarities of the research protocols including adequate follow-up duration, using similar CVD events definition, similar age range and systematic measurement of CVD risk factors.

The TLGS is a longitudinal study started in 1999 to identify risk factors of non-communicable diseases among population in district no.13 of Tehran, the capital of Iran. The study methods have been described elsewhere [23,28].

## Statistical analysis

### Developing of Non-laboratory-based model

In our previously reported risk model based on laboratory risk factors, we considered age, sex, systolic blood pressure (SBP), total cholesterol (TC), diabetes (based on fasting blood test), smoking status, family history of CVD, and waist to hip ratio (WHR) [22]. In current study, we developed a new non-laboratory-based model and named it as simplified PARS (SPARS) by considering risk factors like: age, smoking status, SBP, self-reporting history of diabetes, and WHR. We used Cox proportional hazards regression to estimate the hazard ratios (HR). Deviation from the proportional hazards assumption was assessed graphically and by fitting an extended Cox model, including time-varying covariates. There was no significant deviation from the proportional hazards assumption. Then we constructed a 10-year simplified risk assessment chart of CVD incidence using the SPARS model. SBP was categorized into four classes based on Joint National Committee on Prevention, Detection, Evaluation, and Treatment of High Blood Pressure (JNC 7) [29]: <120, 120–139, 140–159, and ≥160 mm Hg. Waist-to-hip ratio (WHR) was categorized as <0.85, 0.85–0.90, 0.90–0.95 and >=0.95 in females and <1, 1–1.05, 1.05–1.10 and >=1.10 in males [30]. Smoking status was categorized as smoker and non-smokers.

The simplified risk chart displays CVD risk thresholds as <5% (low risk), 5% to <10% (intermediate risk), 10% to <15% (high risk) and >15% (very high risk) as a colored chart. The thresholds were chosen conceptually based on ROC curve and indices introduced by Song et al. [31]. Then they are confirmed by some cardiologists about usefulness of them in classifying individuals in low risk to high risk in target population.

The performance and predictive accuracy of models was assessed by discrimination and calibration. To avoid optimism that might result from assessing risk discrimination in the data from which the model was derived, resampling methods including 50000 random-sample bootstrapping and 10-fold cross-validation were used to assess discrimination using Harrell’s C [32]. The calibration of the risk models were assessed in the derivation dataset by Nam-D’Agostino chi-square test [33].

### Evaluation of External validation

External validation assesses whether the model can be used in a population other than the one in which the model was derived [34].

The performance of the non-laboratory-based SPARS prediction function among the TLGS cohort as an external cohort was assessed using two evaluations. First, SPARS function was refitted using the same variables in the equation by applying multiple Cox (proportional hazards) regression model in the TLGS database. Second, recalibration of SPARS function were done using the method applied in the new revised WHO model [10]. The process involved the use of mean risk factor levels (from TLGS) and country-specific estimates of annual incidence of CVD within five-year age groups, from GBD 2017 (Methods.A in the Appendix).

Model performance was evaluated by three steps: comparison of regression coefficients (Methods.A in the Appendix), discrimination using Harrell’s C, and calibration by Nam-D’Agostino chi-square test.

Further, all external validation analysis were also repeated for previous reported laboratory-based model (PARS) to assess external validation of laboratory-based model.

SAS software, version 9.4 (SAS Institute Inc) was used for statistical modeling and analysis. Chart generation and model validations were performed using Matlab version 8.6 (The MathWorks Inc., Natick, MA, USA).

## Results

### Non-laboratory-based model development

During 49452.8 person-years of follow-up (range 0.1–12, median 10.9 years), there were a total of 705 events related to cardiovascular disease (564 IHDs, 141 strokes). Mean age of male and female was 51.2 ± 11.9 and 50.3 ±11.3 years, respectively. There was a higher prevalence of smoking in men than women (41.6 vs. 3.3), while a higher proportion of women had high WHR (94.6 vs. 39.2) and self-reported diabetes (8.6 vs. 5.8). SBP distribution was similar between sexes.

HRs for fatal and non-fatal CVD events for each risk predictor included in the non-laboratory-based model are provided in Table 1 and compared with the laboratory-based model.

**Table 1:** Non- Laboratory-based and laboratory-based models for predicting cardiovascular disease outcomes, Isfahan Cohort Study, 2001–2011.

Risk factors	Estimate	Hazard Ratio	95% CI*

Non-laboratory-based (SPARS)			

Age at baseline per 10 years	0.38227	1.466	1.371–1.566
Male	0.32309	1.381	1.076–1.774
WHR			
Female: <0.85, male: <1			
Female: 0.85-0.90, male: 1–1.05	0.10478	1.11	0.867–1.422
Female: 0.90–0.95, male: 1.05–1.10	0.17986	1.197	0.898–1.595
Female: >=0.95, male: >=1.10	0.2944	1.342	1.012–1.780
SBP (mm Hg)			
<120^a^			
120–139	0.47524	1.608	1.316–1.966
140–159	0.79045	2.204	1.743–2.788
>=160	1.13051	3.097	2.405–3.989
Self-reported Diabetes	0.73001	2.075	1.697–2.537
Smoking	0.2656	1.304	1.079–1.577
Harrell’s C (95% CI):0.73 (0.71–0.74) Nam-D’Agostino χ^2^:11.01 (p = 0.27).			
**Laboratory-based (PARS)^‡^**			

Age at baseline per 10 years	0.376	1.456	1.361–1.558
Male	0.28957	1.335	1.111–1.508
TC (mg/dl)			
<150^a^	–	–	
150–200	0.20759	1.231	0.879–1.723
200–250	0.34201	1.408	1.013–1.957
250–300	0.45316	1.573	1.113–2.225
>300	0.54847	1.731	1.172–2.556
SBP (mm Hg)			
<120^a^	–	–	
120–139	0.45643	1.578	1.291–1.929
140–159	0.73697	2.09	1.651–2.644
>=160	1.0467	2.848	2.207–3.676
Diabetes	0.63041	1.878	1.570–2.247
High WHR^b^	0.26989	1.31	1.072–1.601
FH of CVD	0.40182	1.495	1.116–2.002
Smoking	0.28974	1.336	1.104–1.617
Harrell’s C (95% CI): 0.73 (0.71–0.75) Nam-D’Agostino χ^2^: 10.82 (p = 0.29)			

Abbreviations: CI, confidence interval; CVD, cardiovascular Disease; TC, total cholesterol; SBP, systolic blood pressure; FH, family history; WHR, waist to hip ratio. ^a^ Reference category. ^b^ WHR ≥ 0.95 in men and ≥ 0.8 in women was considered as a high WHR. ^*^ 95% CI for HR. ^‡^ PARS risk model, published previously by Sarrafzadegan et al. [22].

**Figure 1:**
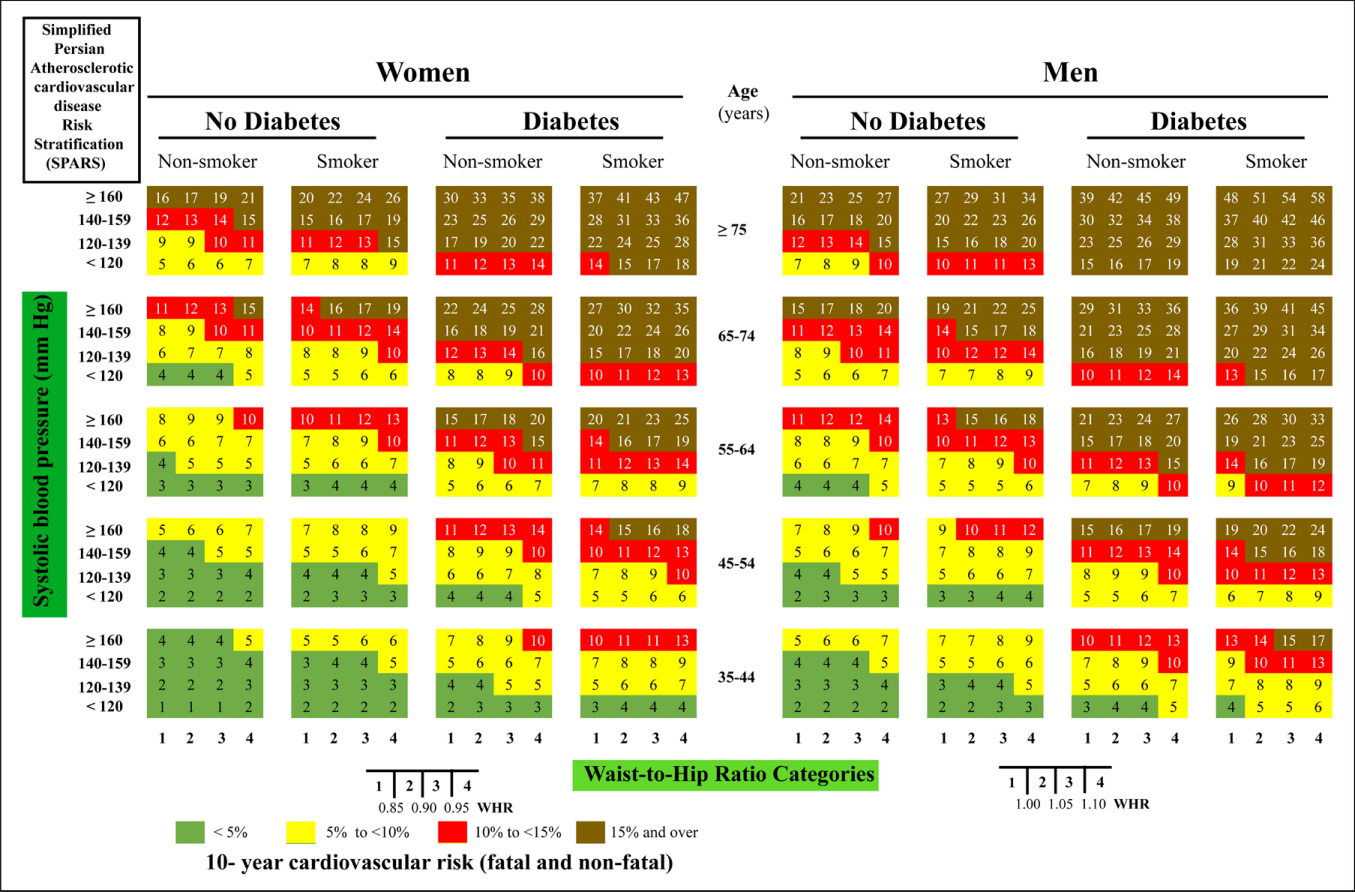
SPARS chart for prediction of 10-year risk of fatal and non-fatal cardiovascular disease in ICS population, 2001–2011.

The 10-fold cross-validation yielded a mean Harrell’s C: 0.73 (95% confidence interval [CI], 0.71–0.74). In bootstrap validation, the mean Harrell’s C was 0.73 (min-max; 0.70–0.77). The Nam-D’Agostino calibration χ^2^ was 11.01 (p = 0.27). Thus, the proposed non-laboratory-based model could be internally validated and has good performance in terms of discrimination and calibration. Fitting new non-laboratory WHO model on our population resulted in C-index of 0.73 (95% [CI], 0.71–0.76) and χ^2^ of 15.76 (p = 0.07) for female and C-index of 0.71 (95% [CI], 0.68–0.74) and χ^2^ of 5.91 (p = 0.75) for male (Table A.1 in the Appendix). Thus, our proposed model performed better than new WHO model. After that, the risk chart based on the proposed non-laboratory-based model was created (Figure 1).

We observed no difference in the Harrell C and a small one in the calibration χ^2^ when we compared non-laboratory-based with the laboratory-based, reported previously (Harrell’s C: 0.73, Nam-D’Agostino χ^2^:10.82; p = 0.29). In addition, we found strong agreement between risk predictions based on laboratory and non-laboratory models. Of individuals at greater than 10% risk using the laboratory-based model, all were also identified as being at greater than 5% risk with the non-laboratory-based model. When using a 10% and 15% risk threshold with the non-laboratory-based model, about 93% and 71% of CVD events respectively were identified.

**Figure 2:**
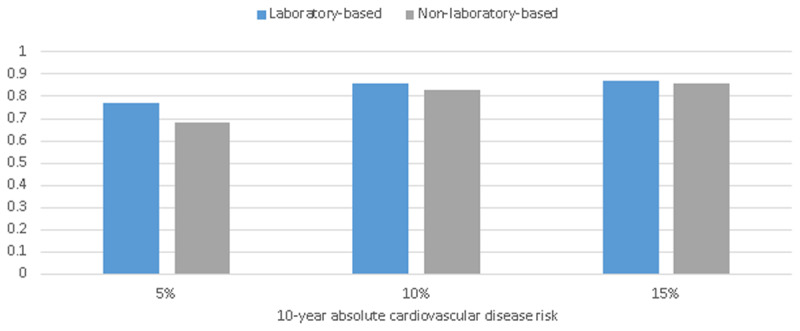
Patients correctly classified as a high or low risk at various cutoff levels of 10-year risk of cardiovascular disease.

In spite of reporting C statistics as suitable criteria for the overall predictive discrimination of the risk models, since the most clinical policy regarding treatment are often considered a specific absolute level of risk, it is useful to illustrate if the risk models classified patients correctly at different levels of 10-year CVD risk, commonly used in guidelines [20]. One measure is the percentage of individuals correctly classified as the sum of the number of true negatives (those below the risk threshold without any CVD events) and the true positives (those who are above the risk threshold and ultimately did have an event) divided by the total number of individuals in the sample (Figure 2, Table 2). The laboratory-based and non-laboratory-based models classified individuals at the same rates across the commonly used risk levels in clinical guidelines. Both laboratory-based and non-laboratory-based models correctly classified over 80% of patients when the threshold for risk was set at 10% and 15%. This percentage was reduced as the risk threshold dropped, and correctly classified 77% and 68% of patients at 5% risk based on laboratory-based and non-laboratory-based models, respectively.

**Table 2:** True positive and true negative based on PARS and SPARS models across different risk thresholds.

Thresholds	PARS		SPARS	

	True positive	True negative	True positive	True negative

5%	50.1% (353/705)	81.3% (3844/4727)	66.2% (467/705)	68.1 (3217/4727)
10%	17.6% (124/705)	96.5% (4561/4727)	31.6% (223/705)	90.4% (4273/4727)
15%	5.2% (37/705)	98.9% (4676/4727)	13.9% (98/705)	96.7% (4573/4727)

### External validation

A total of 824 CVD events occurred during 55982.8 person-years of follow-up in TLGS cohort. The 10-year CVD event rates were 1.4% in the ICS and 1.5% in the TLGS. Numbers of participants, person-years of follow-up, CVD events, and the risk factor levels at the baseline examination of the two cohorts are shown in Table 3.

**Table 3:** Baseline Risk Factors, Person-Years of Follow-up, and CVD events in the ICS and TLGS Cohorts.

	ICS*						TLGS					

	Men (N = 2648)			Women (N = 2784)			Men (N = 2306)			Women (N = 2976)		

	Participants WithRisk Factor, %	Person-years of follow-up	CVD events	Participants WithRisk Factor, %	Person-years of follow-up	CVD events	Participants WithRisk Factor, %	Person-years of follow-up	CVD events	Participants WithRisk Factor, %	Person-years of follow-up	CVD events

Total		23931	379		25522	326		23812.9	464		32169.9	360
TC (mg/dl)												
<150	11.4	2826.4	27	7.5	1917	15	5.3	1294.2	17	3	991.4	2
150–200	35	8507.1	112	30.4	7938.5	71	34.4	8375.3	125	26.9	8999	39
200–250	34.1	8144.3	131	36.4	9206.3	123	42.2	10136.2	193	40.9	13156.3	148
250–300	14.2	3253.8	79	18.6	4710.25	77	15.1	3369.5	104	22.3	7036.2	112
>300	5.2	1198.9	30	7.2	1749.8	40	3	637.6	25	6.9	1986.9	59
SBP(mm/Hg)												
<120	44.4	11132.3	96	44.9	11652	67	48	11988.3	141	45.3	15165	85
120–139	37.1	8741.5	148	33.7	8746.2	116	33.9	8038	162	33.5	10835.4	105
140–159	12	2683	76	13.4	3347.7	78	12.6	2699.7	102	14.5	4337.4	105
>=160	6.6	1374.1	59	8	1776.1	65	5.6	1086.9	59	6.7	1832.1	65
Diabetes												
Laboratory-based	9.3	2059.8	82	12.6	3040.7	87	15.4	3009.7	142	17.8	5022.5	155
Self-reporting	5.8	1289.8	51	8.6	1984.4	69	10.7	2085.3	96	12.5	3480.3	123
Smoker	41.6	9809.1	159	3.3	805.3	18	27.6	6487.9	120	3.8	1188.6	14
FH of CVD	5	1205.7	22	5.7	1415.8	27	15.6	3618.3	83	19.9	6188.4	101
WHR												
1	86.3	20719.7	299	13.8	3673.1	25	79.1	19236.2	306	39.8	13312.5	61
2	9.3	2236.6	49	16.4	4103.2	44	15.8	3557.3	107	21.6	7010.7	77
3	3.3	714.1	25	24.4	6272.7	71	4.2	841.3	41	20.8	6590.4	97
4	1.1	260.5	6	45.4	11472.9	186	0.9	178.1	10	17.8	5256.2	125

Abbreviations: ICS, Isfahan Cohort Study; TLGS, Tehran Lipid and Glucose Study; CVD, cardiovascular Disease; TC, total cholesterol; SBP, systolic blood pressure; FH, family history; WHR, waist to hip ratio. * Data for the ICS risk factors are from Sarrafzadegan et al. [22].

The HRs for major CVD risk factors based on non-laboratory-based risk function were obtained from Cox regression model for TLGS cohort and compared with original functions that were developed on ICS cohort (Table 4). Major risk factors showed a similar relation to CVD in both cohorts for non-laboratory model. For most risk factor categories, the magnitude of the HRs did not differ significantly. The results for the non-laboratory-based model showed few exceptions that reached statistical significance, the significant higher HR for men (P = 0.005) and WHR (P = 0.04, 0.03 for the 3^rd^ and 4^th^ category, respectively) in TLGS.

**Table 4:** Relative risk and performance comparisons of ICS and TLGS based on non-laboratory SPARS risk function.

Risk factors	ICS		TLGS	

	Estimate	Hazard Ratio(95% CI)	Estimate	Hazard Ratio(95% CI)

Age	0.03823	1.039 (1.032–1.046)	0.04494	1.046 (1.039–1.053)
Male^†^	0.32309	1.38 (1.076–1.774)	0.76677	2.153 (1.801–2.573)
WHR				
1			–	–
2	0.10478	1.11 (0.867–1.422)	0.35549	1.427 (1.188–1.713)
3^†^	0.17986	1.197 (0.898–1.595)	0.55846	1.748 (1.399–2.184)
4^†^	0.2944	1.342 (1.012–1.780)	0.69904	2.012 (1.577–2.567)
SBP (mm/Hg)				
<120^a^			–	–
120-139	0.47524	1.608 (1.316–1.966)	0.21399	1.239 (1.029–1.490)
140-159	0.79045	2.204 (1.743–2.788)	0.70242	2.019 (1.643–2.481)
>=160	1.13051	3.097 (2.405–3.989)	0.84719	2.333 (1.825–2.982)
History of Diabetes	0.73001	2.075 (1.697–2.537)	0.88382	2.42 (2.064–2.837)
Smoking	0.2656	1.304 (1.079–1.577)	0.47038	1.601 (1.308–1.959)
	Harrell’s C (95% CI): 0.73 (0.71–0.74) Nam–D’Agostino χ^2^: 11.01 (p = 0.27)		Harrell’s C (95% CI): 0.77 (0.75–0.78) Nam–D’Agostino: 29.89 (p = 0.001)	

Abbreviations: ICS, Isfahan Cohort Study; TLGS, Tehran Lipid and Glucose Study; CVD, cardiovascular Disease; SBP, systolic blood pressure; WHR, waist to hip ratio. ^†^ Hazard ratio in the ICS is significantly different from that in the TLGS (*P*-value < 0.05).

For the laboratory-based model, male gender was associated with a higher HR (P < 0.001), and SBP of 120 to 139 mm/Hg was associated with lower HR (P = 0.03) in TLGS. Other risk factors showed a similar relation to CVD in both cohorts (Table 5).

**Table 5:** Relative risk and performance comparisons of ICS and TLGS based on laboratory-based PARS risk function.

Risk factors	ICS^*^		TLGS	

	Estimate	Hazard Ratio(95% CI)	Estimate	Hazard Ratio(95% CI)

Age	0.03759	1.038 (1.031–1.045)	0.04592	1.047 (1.04–1.054)
Male ^†^	0.28957	1.335 (1.111–1.508)	0.71764	2.05 (1.748–2.403)
TC (mg/dl)				
<150^a^	–	–	–	–
150–200	0.20759	1.231 (0.879–1.723)	0.00956	1.01 (0.627–1.625)
200–250	0.34201	1.408 (1.013–1.957)	0.41855	1.52 (0.956–2.417)
250–300	0.45316	1.573 (1.113–2.225)	0.55737	1.746 (1.087–2.804)
>300	0.54847	1.731 (1.172–2.556)	0.95743	2.605 (1.571–4.321)
SBP (mm/Hg)				
<120^a^	–	–	–	–
120–139^†^	0.45643	1.578 (1.291–1.929)	0.15221	1.164 (0.967–1.402)
140–159	0.73697	2.09 (1.651–2.644)	0.60783	1.836 (1.493–2.259)
>=160	1.0467	2.848 (2.207–3.676)	0.74208	2.1 (1.643–2.684)
Diabetes	0.63041	1.878 (1.570–2.247)	0.79142	2.207 (1.899–2.564)
High WHR	0.26989	1.31 (1.072–1.601)	0.31902	1.376 (1.154–1.640)
FH of CVD	0.40182	1.495 (1.116–2.002)	0.38066	1.463 (1.240–1.727)
Smoking	0.28974	1.336 (1.104–1.617)	0.49554	1.641 (1.341–2.010)
	Harrell’s C (95% CI): 0.73 (0.71–0.75) Nam–D’Agostino χ^2^: 10.82 (p = 0.29)		Harrell’s C (95% CI): 0.78 (0.76–0.79) Nam–D’Agostino χ^2^: 28.57 (p = 0.001)	

Abbreviations: ICS, Isfahan Cohort Study; TLGS, Tehran Lipid and Glucose Study; CVD, cardiovascular Disease; TC, total cholesterol; SBP, systolic blood pressure; FH, family history; WHR, waist to hip ratio. * Data for the ICS risk factors are from Sarrafzadegan et al. [22]. ^†^ Hazard ratio in the ICS is significantly different from that in the TLGS (*P*-value < 0.05).

**Figure 3:**
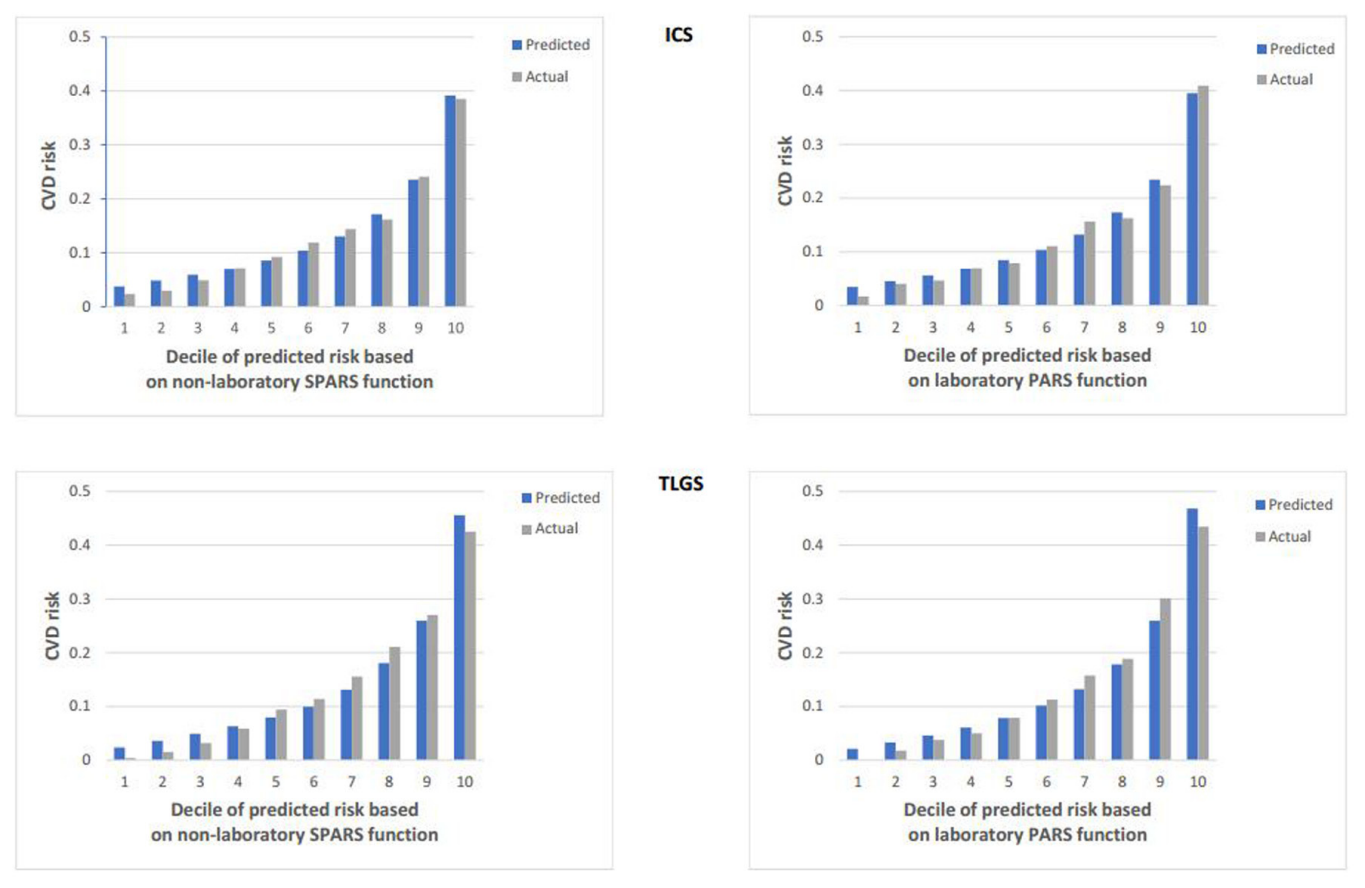
Actual and predicted cardiovascular disease events by deciles of risk for the laboratory and non-laboratory-based model in ICS and TLGS.

In the discrimination analysis, non-laboratory-based model separated individuals with CVD events from ones without CVD in the TLGS cohort nearly as well as in the ICS cohort. The Harrell’s C was 0.77 (95% CI, 0.75-0.78) for non-laboratory-based model in TLGS versus 0.73 (95% CI, 0.71–0.74) in ICS. However, with respect to calibration, non-laboratory-based model slightly overestimated the event rates observed in the TLGS cohort. The Nam-D’Agostino χ^2^ was 29.89 (p = 0.001) in TLGS versus 11.01 (p = 0.27) in ICS (Figure 3). In the recalibrated non-laboratory-based function, the C statistic values was 0.73 (95% CI, 0.71–0.75). Thus, the model retained acceptable discrimination performance in the external validation cohort.

The same results were obtained for laboratory-based model (Harrell’s C: 0.78, 95% CI: 0.76–0.79 in TLGS versus 0.73, 95% CI: 0.71–0.75 in ICS; Nam-D’Agostino χ^2^: 28.57, p = 0.001 in TLGS versus 10.82, p = 0.29 in ICS; C statistic: 0.74, 95% CI, 0.72–0.76).

## Discussion

We developed a ‘Simplified’ non-laboratory based CVD risk chart and named it SPARS, then evaluated and validated both PARS laboratory-based, published previously [22], and SPARS risk prediction models with TLGS as an external cohort.

The proposed SPARS model does not require laboratory measurements such as serum lipids or glucose. This simplified non-laboratory-based model which is clinically convenient, user-friendly, and affordable, can be applicable in situations with little access to laboratory tests, particularly in primary health care systems (PHC) or in low-resource settings like health houses that cover the rural population in Iran [35]. Our study shows that the non-laboratory-based risk model can predict CVD outcomes with similar accuracy to a laboratory-based one. Our values of predictive discrimination of 0.73 for the non-laboratory-based model are not different from the corresponding values in the laboratory-based model. We also found strong agreement with the laboratory-based model with respect to the classification of patients to moderate or high risk group, similar to findings from previous reports [10,18,20]. Furthermore, our study showed that 90% of patients with diabetes, who were classified as being at greater than 10% risk of developing CVD in 10 years based on the PARS model, were also classified as being at greater than 10% risk with the SPARS model. Other studies reported poor performance among people with diabetes (e.g., 45% of men and 25% of women in WHO study) [10,18]. It might be due to the inclusion of self-reported history of diabetes in our non-laboratory-based model that highlights the importance of including a diagnosis of diabetes in the risk score. Therefore, because of the ability of non-laboratory screening tools to correctly classify patients at the thresholds recommended by guidelines for initiating treatment, these tools are suitable to predict CVD risk in LMICs to reduce the cost regarding laboratory tests.

We developed SPARS in an analogous manner to the previous reported PARS model to provide user-friendly chart. Nevertheless, the color code has been revised to facilitate application compared with those of previous PARS model. Our chart differs from the new WHO chart, in that it includes waist to hip ratio instead of the body-mass index (BMI). In both PARS and SPARS models, WHR was a significant predictor of cardiovascular risk in our population.

Our user-friendly chart takes into account features of practicality, cost, and feasibility. Health workers can determine the risk value and treatment can be initiated in one clinic visit with minimum equipment, and less cost and time needed as there is no waiting time for laboratory results. Major global guidelines promote the use of multivariable risk models to guide treatment decisions. Decisions about whether to initiate pharmacological treatment in addition to lifestyle behaviors interventions as well as treatment intensity are guided by the level of the risk of these models. Considering risk assessment models in guidelines recommendations can be seen in many guidelines like hypertension or hyperlipidemia. Individuals at higher risk for CVD events require more intensive management. Conversely, low-risk individuals can be spared from the associated harms and high costs of overtreatment. The World Health Organization (WHO) has also recommended two packages of essential NCD interventions (WHO-PEN and HEARTS) with protocols that include simple and affordable tools such as CVD risk assessment charts for early detection and treatment [7].

Moreover, in many LMICs, clinical guidelines are developed based on laboratory-based risk assessment models which are costly and guideline developers do not consider the availability of facilities or health-care workers to implement these laboratory-based screening or treatment guidelines [20].

In the present study, PARS and SPARS prediction models were evaluated externally using data obtained from the TLGS study. HRs for major CVD risk factors were remarkably similar to those derived from the ICS, except, the HRs for males and for WHR were somewhat higher in TLGS. Both the original and the recalibrated PARS and SPARS functions discriminated well between individuals with CVD and without CVD in ICS and the TLGS. In the calibration analysis, slight overestimation was observed when the PARS and SPARS functions were applied directly to the TLGS. The calibration is generally poor, mainly owing to the differences in the relative risks associated with risk factors and the mean levels of the risk factors between the two cohorts [36]. To use a risk assessment tool optimally and to be acceptable for treatment guidelines, clinicians need to be confident that the absolute risk prediction functions can be generalized to other settings beyond where they were originally developed [36]. We have demonstrated that both PARS and SPARS prediction functions work reasonably well among the TLGS population.

To overcome over-estimation, we recalibrated both PARS and SPARS risk models based on national and more contemporary statistics from GBD applying new approach used in WHO models [10]. This approach involved few modeling steps [17,37,38]. Descriptive epidemiological data, including country-specific cardiovascular disease incidence to reflect changes in disease incidences and risk factor profiles can be readily incorporated to revise models [10].

Recently, the WHO developed laboratory and non-laboratory risk charts, however they were only presented for regions and not for individual countries, although CVD risk differs between countries within some regions [39]. The risk chart developed in our study could be particularly useful for application in other countries in the EMR or other LMICs because many of these countries do not have locally developed risk scores based on their own cohort studies.

We also developed electronic and mobile application based on the SPARS risk assessment chart for easier use by general practitioners, physicians with related specialties like cardiologists and other health workers. All can rapidly implement a simple non-laboratory approach for initial screening, preventive, and treatment interventions without need for blood testing. Web-based program are freely accessible at http://www.prognosis.ir/Pars/index2.php. Other electronic and mobile application are under preparation to be used by the general public and provide them preventive recommendations.

Our study has several points of strength. First feature is the development of practical model merely based on non-laboratory measurement. Such simplified approaches could be used as part of stepwise approaches to help target laboratory testing in people most likely to benefit from the extra information and used even when values for some risk factors are unavailable for individuals in low-resource settings. A second is the recalibration approach we have used. It involves the use of GBD statistics. So, it can be easily developed and require fewer modelling steps. Third feature is that the risk model reported here can predict combined outcome of fatal and non-fatal events, thereby improving on risk calculators that predict fatal events alone. Predicting only fatal events in risk models significantly underestimates total CVD risk, particularly in populations with a low fatality rate [18].

However, our study has some limitations. First, we did not include heart failure, vascular dementia, or peripheral vascular disease as outcomes, thus the overall cardiovascular risk may be underestimated, however, only few risk assessment models include these outcomes. Second, as in all cohort studies, some lost to follow-up participants was inevitable. The main reason for loss to follow-up in the ICS was related to phone number changes in some city blocks, which was done by the national government to develop and expand communication infrastructure. There were also some changes in address and only a few people were not willing to take part in multiple follow-ups. Therefore, loss to follow-up was likely to be largely random [22,25,27]. Finally, although the current study has broad coverage in comparison with other similar studies in Iran, recruitment was still limited to the central area of Iran.

In conclusion, we derived a non-laboratory-based cardiovascular risk prediction model using age, sex, smoking status, a self-reporting history of diabetes, blood pressure levels, and waist to hip ratio, all of which can be obtained in one outpatient visit. Such a user-friendly tool can help primary health care providers to determine the risk of cardiovascular disease (CVD) using an affordable screening tool. Remarkably, the simplified model was in close agreement with the laboratory-based model. We also validated and recalibrated both laboratory-based and non-laboratory-based models using mean risk factor levels based on the external cohort and country-specific estimates of annual incidence of CVD from GBD statistics, thereby enabling more accurate identification of individuals at high risk of cardiovascular disease in different settings.

## Additional File

The additional file for this article can be found as follows:

10.5334/gh.890.s1 Appendix. Methods A.
