# Supplementary material for: Developing Non-Laboratory Cardiovascular Risk Assessment Charts and Validating Laboratory and Non-Laboratory-Based Models
Source: Glob Heart. 2021 Sep 2;16(1):58. doi: 10.5334/gh.890 (PMC8428313; doi:10.5334/gh.890)
Supplement: Appendix. — Methods A. [file gh-16-1-890-s1.pdf]

Appendix

Methods. A

Methods used for recalibration

We used the β coefficients of the original PARS and SPARS models to estimate 10-year predicted risk of CVD for each of the age groups using the mean risk factor values of target population (*x*) by

$$p_{pred.agegroup} = 1 - {S_0}^{exp(\sum \beta x - M)}.$$

Where, *M* are values at which each risk factor was centered in PARS and SPARS model and S_0 is baseline survival from PARS and SPARS models.

Then, expected 10-year risk was calculated based on annual incidence using following formula:

$$p_{expe.agegroup} = 1 - \exp(-Incidence \times 10)\ .$$

We then derived recalibration factors by using the intercept and slope of the regression line of transformed expected 10-year risk on predicted risk as following:

$$\ln\left(-\ln\left(1 - p_{expe.\text{agegroup}}\right)\right) = b_1 + b_2 \times \ln(-\ln(1 - p_{pred.\text{agegroup}}).$$

Finally, the SPARS and PARS risk models, rescaled using the recalibration factors, were then used to estimate appropriate risks for each potential risk factor combination,

$$p_{pred} = 1 - {S_0}^{exp(\sum \beta x - M)}$$

$$p_{newpred} = 1 - \exp(-\exp\left(b_1 + b_2 \times \ln\left(-\ln\left(1 - p_{pred.10}\right)\right)\right)).$$

Where, *x* are the risk factor values.

Assessment of regression coefficients in external validity

Equality of regression coefficients was assessed by comparing the hazard ratios in the ICS and TLGS functions using z statistics as follow: $z = (b_{ICS} - b_{TLGS})/\sqrt{SE_{b_{ICS}}^2 + SE_{b_{TLGS}}^2}$, where b_{ICS}

and b_{TLGS} are the β coefficients of the ICS and TLGS model, respectively with $SE_{b_{ICS}}$ and $SE_{b_{TLGS}}$, as the standard errors (SEs) of b_{ICS} and b_{TLGS}.

Table A.1: WHO risk model tested in ICS data

	Men				Women			
	Main effect		Age interaction term		Main effect		Age interaction term	
**Laboratory-based models**	HR	P-value	HR	P-value	HR	P-value	HR	P-value
Age at baseline per 5 years	2.12	<.0001			1.48	0.02		
Current smoking status	2.15	0.1522	0.963	0.4289	0.195	0.50	1.168	0.42
Systolic blood pressure per 20 mm Hg	2.657	<.0001	0.945	0.0033	1.71	0.02	0.982	0.38
History of diabetes	2.125	0.2938	0.991	0.8771	2.22	0.26	0.989	0.85
Total cholesterol per 1 mmol/L	3.782	0.0041	0.916	0.0271	1.62	0.39	0.976	0.61
C-Statistic (95% CI)	0.73(0.70-0.75)				0.73(0.71-0.77)			
χ^2	$6.24\ (p = 0.72)$				$4.18\ (p = 0.90)$			
**Non-laboratory-based models**								
Age at baseline per 5 years	1.79	0.001			1.66	0.006		
Current smoking status	2.245	0.13	0.961	0.40	0.11	0.35	1.22	0.29
Systolic blood pressure per 20 mm Hg	2.90	<.0001	0.94	0.001	1.80	0.009	0.979	0.27
BMI per 1 kg/m²	1.02	0.80	1	0.88	1.09	0.14	0.996	0.38
C-Statistic (95% CI)	0.71(0.68-0.74)				0.73(0.71-0.76)			
χ^2	$5.91\ (p = 0.75)$				$15.76\ (p = 0.07)$			
